# Induction of PGC-1α Expression Can Be Detected in Blood Samples of Patients with ST-Segment Elevation Acute Myocardial Infarction

**DOI:** 10.1371/journal.pone.0026913

**Published:** 2011-11-07

**Authors:** Óscar Fabregat-Andrés, Alberto Tierrez, Manuel Mata, Jordi Estornell-Erill, Francisco Ridocci-Soriano, María Monsalve

**Affiliations:** 1 Servicio de Cardiología, Consorcio Hospital General Universitario de Valencia, Valencia, Spain; 2 Fundación Centro Nacional de Investigaciones Cardiovasculares Carlos III, Madrid, Spain; 3 Fundación Hospital General de Valencia, Valencia, Spain; 4 Unidad de TAC y RMN, Eresa, Consorcio Hospital General Universitario de Valencia, Valencia, Spain; 5 Instituto de Investigaciones Biológicas Alberto Sols (CSIC), Madrid, Spain; University of Valencia, Spain

## Abstract

Following acute myocardial infarction (MI), cardiomyocyte survival depends on its mitochondrial oxidative capacity. Cell death is normally followed by activation of the immune system. Peroxisome proliferator activated receptor γ-coactivator 1α (PGC-1α) is a transcriptional coactivator and a master regulator of cardiac oxidative metabolism. PGC-1α is induced by hypoxia and facilitates the recovery of the contractile capacity of the cardiac muscle following an artery ligation procedure. We hypothesized that PGC-1α activity could serve as a good molecular marker of cardiac recovery after a coronary event. The objective of the present study was to monitor the levels of PGC-1α following an ST-segment elevation acute myocardial infarction (STEMI) episode in blood samples of the affected patients. Analysis of blood mononuclear cells from human patients following an STEMI showed that PGC-1α expression was increased and the level of induction correlated with the infarct size. Infarct size was determined by LGE-CMR (late gadolinium enhancement on cardiac magnetic resonance), used to estimate the percentage of necrotic area. Cardiac markers, maximum creatine kinase (CK-MB) and Troponin I (TnI) levels, left ventricular ejection function (LVEF) and regional wall motion abnormalities (RWMA) as determined by echocardiography were also used to monitor cardiac injury. We also found that PGC-1α is present and active in mouse lymphocytes where its expression is induced upon activation and can be detected in the nuclear fraction of blood samples. These results support the notion that induction of PGC-1α expression can be part of the recovery response to an STEMI and could serve as a prognosis factor of cardiac recovery.

## Introduction

Acute myocardial infarction (MI) is the leading cause of death of patients with cardiovascular disease [1], [2]. Left ventricular (LV) dilatation and pump failure following MI are the main causes for poor clinical outcomes. Clinical studies have consistently demonstrated that development of congestive heart failure typically depends on infarct and LV ischemic size areas. MI ischemic process initiates a cascade of progressive structural and geometric changes in the left ventricle, a process commonly referred to as remodeling. The molecular and cellular changes associated with ventricular remodeling affect both the necrotic zone and the non-infarcted segments of the ventricle and manifest clinically as chamber dilation, ventricular remodeling with increased sphericity of the ventricle and worsened cardiac function, greatly limiting patients living conditions [3], [4].

MI recovery thus depends on two clearly distinct but related factors. The first one is the capacity of cardiomyocytes to survive the ischemia-reperfusion (IR) injury, and the second one is the intrinsic regenerative capacity of the damaged tissue, that depend both on the patient genetics but also on its life-style and clinical history. Importantly, not only cardiomyocytes but several other cell types have been proposed to play a role in the repair/regeneration process and impact, both positively and negatively, the final outcome [5]. For example, early activation of the inflammatory system protects from ventricular remodeling although its sustained activation favors it. Evidence suggests that T cells play a significant role in controlling the post-infarct inflammatory response [6]. Their behavior is likely to depend to a large extent on the capacity of the damaged tissue of recovering its oxidative capacity. Low oxygen tension activates critical transcription factors like HIF (*Hypoxia Induced Factor*) that facilitates cell survival by shifting the cellular metabolism from oxidative to glycolytic [7]. If this metabolic shift were not reversed during the “recovery-regenerative” phase the result would be increased cardiac overload and failure [8]. The crucial regulatory factors/pathways that mediate this metabolic shift are still only partially elucidated [9].

Peroxisome proliferator activated receptor γ coactivator -1α (PGC-1α) is a transcriptional coactivator and a master regulator of genes involved in oxidative metabolism and mitochondrial biogenesis [10] that plays a key role in the metabolic control in the cardiac muscle [11] and participates in cardiomyocyte differentiation [12]. Importantly PGC-1α knock out mice develop ventricular dysfunction and are prone to cardiac failure following transverse aortic constriction [13]. Accordingly, PGC-1α levels are reduced in the heart following MI by coronary artery ligation in rats [14] while treatment with angiotensin II receptor blockers (ARB) and PPAR agonists (pioglitazone and rosiglitazone), preserve both ventricular function and PGC-1α levels, and have been demonstrated to attenuate myocardial ischemia-reperfusion injury [15], [16], [17]. Another study using rats demonstrated that the gene expression of PGC-1α was also down-regulated in the heart in congestive heart failure [18].

All these results support the notion that while severe hypoxia activates HIF [19] and down-regulates PGC-1α levels in the cardiomyocyte, PGC-1α induction is likely to be crucial during the regenerative phase to facilitate the recovery of the cardiac muscle oxidative capacity [20]. Therefore, monitorization of the patient's capacity to induce PGC-1α expression post-infarction would be of potential use to pre-evaluate its later recovery, however that monitorization could only be feasible if PGC-1α induction post-infarction could be detected in the nuclear fraction of blood samples. In this respect PGC-1α has been previously reported to be present in circulating neutrophils and lymphocytes [21].

In summary, from what we know so far from PGC-1α biology we think that the capacity of a tissue to induce PGC-1α after an hypoxic event, such as a MI, could predict the regenerative capacity of the tissue. In the particular case of the human heart following a MI, that predictive value is both particularly relevant and hard to assess directly, because we cannot take biopsies of the infarcted heart in order to measure PGC-1α levels. Hence, the potential use of PGC-1α as a predictor of a patient's recovery capacity would have no translational use unless we showed first that we could detect PGC-1α induction in circulating immune cells of infarcted patients.

Therefore, we decided to test if PGC-1α could be detected in human blood mononuclear cells and if its expression was induced following lymphocyte activation post-infarction.

Our results show that PGC-1α is present and active in both B and T lymphocytes, and its expression is activated upon lymphocyte activation. More importantly, PGC-1α induction can be detected in blood mononuclear cells from MI patients, 72 hours (h) post-infarction, with PGC-1α induction correlating well with the size of the hypoxic area.

## Materials and Methods

### Humans

This is a cohort study of 38 patients with confirmed ST-segment elevation myocardial infarction (STEMI) that were subjected to a reperfusion strategy and discharged alive from Coronary Care Unit of the “Consorcio Hospital General Universitario de Valencia” (CHGUV), Spain. The patients were admitted in the period from March-2009 through March-2010. The diagnosis of STEMI was based on the definition of the European Society of Cardiology (ESC) Guidelines [22], [23], by the presence of clinically appropriate symptoms (ischemic characteristic chest pain and/or autonomic nervous system activation), increased blood levels of biomarkers troponin I (TnI) and creatin-kinase MB (CK-MB) and persistent ST-segment elevation on the electrocardiogram (ECG). Reperfusion therapy included mechanical and pharmacological procedures, such as fibrinolytic treatment, primary percutaneous coronary intervention (PCI) and rescue PCI after failed pharmacological reperfusion. For exclusion criteria see Supp. Info. Blood samples were drawn on admission to the hospital and 72 h later. Total mRNA and protein were isolated from the blood mononuclear fraction.

Infarct size was estimated from the determination of the necrotic area by late gadolinium enhancement (LGE) in cardiac magnetic resonance (CMR) studies. Maximun CK-MB and TnI plasma levels, left ventricular ejection function (LVEF) and regional wall motion abnormalities (RWMA) were also used as markers of cardiac injury.

### Patient exclusion criteria

Were excluded patients with previous history of coronary artery disease, in order to avoid bias measuring myocardial necrosis images obtained by CMR. We also excluded patients with chest pain latency longer that 150 minutes, to avoid miss interpretation of the results obtained from blood samples at the time of hospital admission. Moreover, patients resuscitated, those that suffered a second infarction in the first 72 h post admission, or having their highest CK value on admission, were also not included in the study. The presence of left bundle branch block or atrial fibrillation of ECG at admission was also an exclusion criteria.

*Clinical data collected* included: clinical history including, cardiovascular risk factors and ambulatory treatment, relevant biochemical values, including fasting glucose (mean of three independent values), hemoglobin and hematocrit at admission, leucocytes at admission and 72 h later (including total leucocytes, lymphocytes, monocytes and their relative percentages), CRP at admission, creatinine at admission and the glomerular filtration rate estimated by MDRD equation, lipid profile (including total cholesterol, LDLc, HDLc and triglycerides), glycosilated hemoglobin (Hb1Ac), and the plasma curve of cardiac biomarkers (peak values were used for the analysis), reperfusion procedure used (pharmacological strategy with tenecteplase or mechanical process as primary or recue PCI), echocardiographic data, performed on the first 72 h, with all the M-mode parameters and two dimensional analysis, including LVEF and RWMA, hemodynamic data, including LVEF by ventriculography and coronary arteries affected by the coronarography (considering significant stenosis those with more than 75% of the vessel lumen of epicardial coronary arteries or more than 50% of the main left artery, and CMR data, including the regular determinations of both ventricles, their EF and myocardial mass, the necrotic mass estimated by late gadolinium enhancement (LGE), and the presence of microvascular obstruction).

### Cardiac Magnetic Resonance (CMR) studies

CMR was performed with a 1.5 T unit (Magneton Sonata, Siemens, Erlangen, Germany). Functional images of long-axis and short axis were obtained using ECG-gated SSFP (*Steady State Free Precession*) sequences (8 mm slice thickness with 2-mm gap between short-axis slices). Edema detection was carried out using short-axis black-blood, T2-weighted (short time inversion recovery) in the same views as the cine sequences using a HASTE (*Half-Fourier Acquisition Single-shot Turbo spin Echo*) multisection sequence (RT, 2 R-R intervals; ET, 33 ms; IT, 170 ms; slice thickness, 8 mm; inter-slice interval, 2 mm; flip angle, 160°; matrix, 256×151; bandwidth, 781 Hz/pixel) and a segmented TSE (*Turbo Spin-Echo*) sequence (RT, 2 R-R intervals; ET, 100 ms; IT, 170 ms; slice thickness, 8 mm; interval, 2 mm; flip angle, 180°; matrix, 256×146; bandwidth, 235 Hz/pixel) in case of poor quality with HASTE sequence. Late-gadolinium images were acquired after intravenous injection (0,15 ml/kg) of dimeglubine gadobenate 0.5 M. Non breath-holding ECG-gated single-shot IR-SSFP short-axis in the same views as the cine sequences were performed 1–2 min after contrast administration with fixed long TI (600 ms) to discriminate normal myocardium, infarcted myocardium and microvascular obstruction (MVO). Ten minutes after contrast administration, the same short-axis slices were repeated adjusting the IT to null normal myocardium using non breath-holding single-shot IR-SFP sequences and finally breath-holding 2D and 3D turbo-FLASH (*Fast Low Angle Shot*) sequences. A standard 17-segmented cardiac-model was used for short-used for short-axis slice segmentation and assessing areas of edema and late-gadolinium images. Perfusion defects were assessed visually as persistent deficit during the first–pass contrast. Areas of high T2 signal intensity were identified by visual inspection. Finding of a low-signal-intensity area surrounded by a high-signal-intensity area in these images was considered to indicate an area of MVO. Areas with LGE were also identified by visual inspection, where MVO was defined as an area without signal located within an area of LGE. Transmurality was considered when the area of affected myocardium was >50%. Both edema and LGE were evaluated by manual tracing of these areas, and given values are expressed as percentage of total myocardium mass, as calculated by tracing endocardium and epicardium contours in a dedicated work-station (Argus, Siemens, Erlangen, Germany).

### Mice

C57BL6 PGC-1α^−/−^ mice were originally provided by Dr. Bruce Spiegelman (DFCI, USA) and following embryonic transfer a colony was established within the SPF area of the CNIC animal facility. Male C57BL6 PGC-1α^+/+^ and PGC-1α^−/−^ of 6–8 weeks of age were used.

### Preparation of splenocytes and thymocytes from mice

Spleen and thymus were removed aseptically. Single-cell suspensions were obtained by mechanically disrupting the organs with a syringe plunger in cold PBS supplemented with 2 mM EDTA and 0.1% FBS. Red blood cells were removed incubating the cells in ACK lysis buffer for 5 min at 4°C. Splenocytes and thymocytes were washed in cell culture medium (RPMI 1640 supplemented with 10% FBS, 10 mM HEPES, 2 mM L-Glutamine, 1 mM sodium pyruvate, 1% non essential aminoacids, 100 U/ml penicillin and 100 µg/ml streptomycin) and filtered through a 70 µm nylon cell strainer. Cells were adjusted to a final concentration of 10^6^ cells/ml. Spleen cells were stimulated for 3, 6 and 24 hours with anti-IgM F(ab′)_2_ fragment (1 µg/ml; Jackson immunoresearch).

### Preparation of mononuclear cells from human peripheral blood

4 mL of human peripheral blood collected in K3EDTA Vacutainers (BD) were used to isolate mononuclear cells by Ficoll density gradient centrifugation using Ficoll-Paque™ (Miltenyi Biotec) and following the manufacturer's instructions. Isolated cells were analyzed using Cytospin and Fast Panoptic Staining (Panreac). Only those preparations containing ≥90% of mononuclear cells were used for the analysis.

### Molecular Biology Analysis

Total RNA was isolated using Trizol™ (Invitrogen) and following the manufacturer's instructions. The quality of the RNA was evaluated in a Bioanalyzer and quantitated in a Nanodrop. The RNA used had a ratio of the absorbance at 260 and 280≥1.8 and a RNA integrity number (RIN) ≥8. Relative mRNA expression levels of PGC-1α, cytochrome c (Cyt c) and Mn superoxide dismutase (MnSOD) were determined by quantitative PCR of retro transcribed cDNA (qRT-PCR) with specific primers as previously described [24]. Whole cell extracts were used to analyze protein levels. PGC-1α, Cyt c and MnSOD protein levels in mouse splenocytes were determined by western blot using specific antibodies. The Zeptosens reverse array platform was used to quantify Cyt c protein levels is human samples.

### Mitochondria

Mitochondrial mass was estimated using MitoTracker Green labeling. In short, 2.5×10^5^ splenocytes were incubated in 96-well U- bottom plates with 0.1 µM MitoTracker Green (Invitrogen) in PBS for 30 min at 37°C, washed twice with PBS and fixed with 4% paraformaldehyde for 15 min at 4°C. A FACSCanto™ II cytometer (Becton Dickinson) was used to measure fluorescence intensity of ≥20.000 events per sample. Data was analyzed using the FACSDiva software (Becton Dickinson).

### Ethics

The human experimental protocols were approved by the Institutional Ethical Committee of the Instituto de Salud Carlos III (ISCIII) and the CHGUV (Permit number PI 10/09). All patients signed a written informed consent form. The animal experimental protocols were approved by the Institutional Animal Care and Use Committee of the CNIC (Permit number PA 13/09), and all efforts were made to minimize suffering. All procedures conformed to the Declaration of Helsinki and the NIH guidelines for animal care and use (NIH publication No. 85-23).

### Statistics

Statistical analyses were performed using SPSS for Windows, release 17.0 (SPSS Inc., Chicago, IL). Levene's test for equality of variances and t-test for equality of means were used. All *p*-values refer to two-tailed tests of significance; *p*, 0.05 was considered significant.

## Results

**Figure 1:**
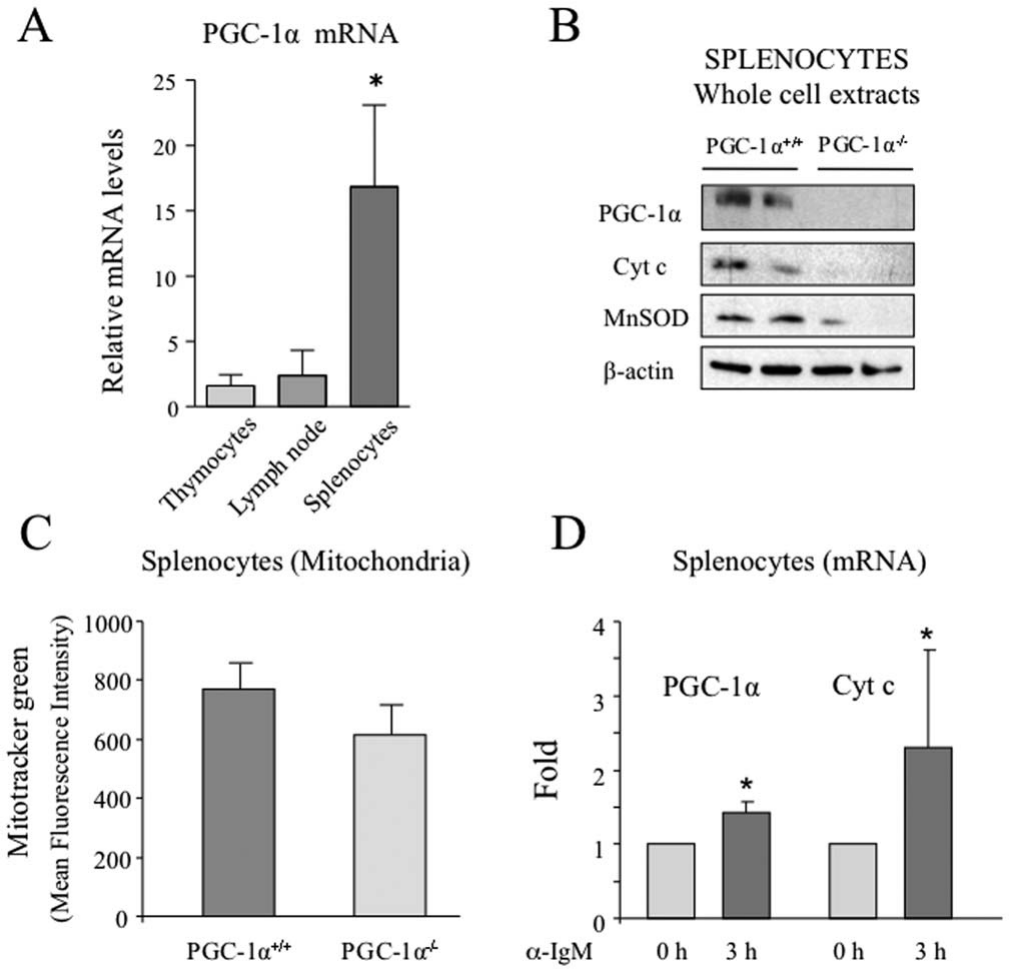
PGC-1α mRNA is induced in mouse splenocytes following stimulation with αIgM. A) PGC-1α is present in mouse splenocytes and tymocytes. Relative PGC-1α mRNA levels mouse splenocytes and tymocytes. B) Whole cell extracts of total splenocytes isolated from PGC-1α^+/+^ and PGC-1α^−/−^ mice analyzed by western blot with specific antibodies against PGC-1α, Cyt c, MnSOD and β-actin as loading control. C) Total splenocytes isolated from PGC-1α^+/+^ and PGC-1α^−/−^ mice were labeled with MitoTracker Green and analyzed by flow cytometry. D) Total splenocytes isolated from C57BL6 mice were stimulated with αIgM for 3 h. Total RNA was extracted and PGC-1α and Cyt c mRNA levels were analyzed by qRT-PCR. Data are means +/−SD. (*) *p*<0.05.

To determine if PGC-1α levels are induced during lymphocyte activation we used a mouse model. First, we evaluated if PGC-1α mRNA could be detected in total splenocyte, thymocytes and lymph nodes from mice. We found that although PGC-1α mRNA was detectable in both preparations PGC-1α levels were about 15 fold higher in splenocytes than in thymocytes and lymph nodes (Fig. 1A). Next, we tested whether PGC-1α protein could be detected in mouse splenocytes. A protein band of the predicted molecular weight could be detected by western blot with a specific antibody (Fig. 1B). In order to asses if this band was in fact PGC-1α, a preparation of total splenocytes from PGC-1α^−/−^ mice was used as negative control. We confirmed that the band was missing from PGC-1α^−/−^ splenocytes and therefore we concluded that PGC-1α is present in mouse splenocytes (Fig. 1B).

To evaluate if the detected PGC-1α protein was playing an active role in the metabolic control of the splenocytes, we tested by western blot the expression levels of well characterized molecular targets of PGC-1α. Cyt c, that is part of the mitochondrial electron transport chain [10], and the detoxification enzyme MnSOD [24] and both were also found to be reduced in the total nuclear fraction of blood samples from PGC-1α^−/−^ mice, suggesting that PGC-1α is not only present, but is active in splenocytes (Fig. 1B).

We further analyzed the mitochondrial content of murine splenocytes from PGC-1α^+/+^ and PGC-1α^−/−^ mice. The cells were labeled with MitoTracker Green, a non-membrane potential-dependent fluorescent marker, and analyzed by flow cytometry. The results show that PGC-1α^−/−^ splenocytes have as an average 20% less mitochondrial content than the PGC-1α^+/+^ splenocytes, indicating that the absence of PGC-1α has a net impact on the number of mitochondria in splenocytes (Fig. 1C).

Finally, we tested if PGC-1α levels were modified upon lymphocyte activation. First we examined the change in total PGC-1α levels upon stimulation of total splenocytes with specific stimuli for B lymphocytes (α-IgM). Splenocytes stimulated with α-IgM showed an increase in both PGC-1α and Cyt c mRNA levels 3 h post stimulation, (Fig. 1D). We concluded that PGC-1α expression is induced upon B cell stimulation. These results support the notion that PGC-1α is present and active in lymphocytes and its levels and activity are induced following lymphocyte stimulation.

**Figure 2:**
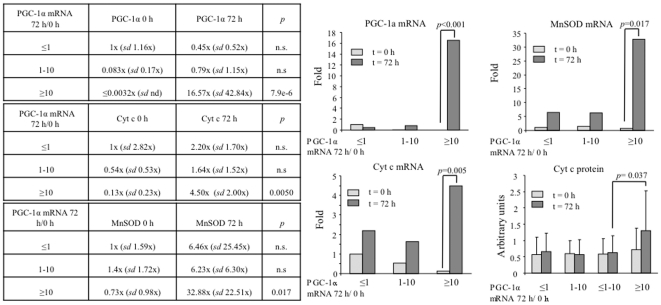
PGC-1α induction after STEMI correlates with Cyt c and MnSOD induction. **A)** PGC-1α mRNA levels, **B)** MnSOD mRNA levels, **C)** Cyt c mRNA levels, **D)** Cyt c protein levels, in the blood mononuclear fraction of 37 STEMI patients right after hospital admission (t = 0) and 72 h later (t = 72 h). Samples were grouped according to the induction rate of PGC-1α mRNA leves after STEMI (PGC-1α mRNA 72 h/PGC-1α mRNA 0 h) in three groups, those that did not induce PGC-1α (≤1), those that induced PGC-1α up to 10-fold (1–10) and those that induced PGC-1α more that 10-fold (≥10). Data are means +/−SD. n.s = non statistically significant. Significance: *p*<0.05.

In order to evaluate if we could detect the induction of PGC-1α expression following STEMI, we isolated total mRNA from blood mononuclear cells, isolated immediately upon admission and 72 h later. PGC-1α levels were monitored by qRT-PCR. We found that the samples segregated in two groups, those that induced PGC-1α and those that did not (≤1/>1). The induction group was further subdivided in those that induced PGC-1α up to 10 fold (1–10), and those that induced it more (≥10) (Fig. 2A). To monitor PGC-1α activity we also determined the induction of the PGC-1α target genes Cyt c and MnSOD. We found that following STEMI not only PGC-1α but also Cyt c and MnSOD mRNA levels were increased (Fig. 2B–C), suggesting that not only PGC-1α mRNA levels are higher, but also its transcriptional activity is elevated following STEMI. This notion is further supported by the observation that the induction of both Cyt c and MnSOD is significantly higher in those patients that induced PGC-1α expression more than 10 fold. To confirm the significance of this observation Cyt c protein levels were directly monitored in whole cell extracts from blood mononuclear cells, and were found to be significantly elevated following STEMI only in the group of patients that induced PGC-1α expression more than 10 fold (Fig. 2D).

**Figure 3:**
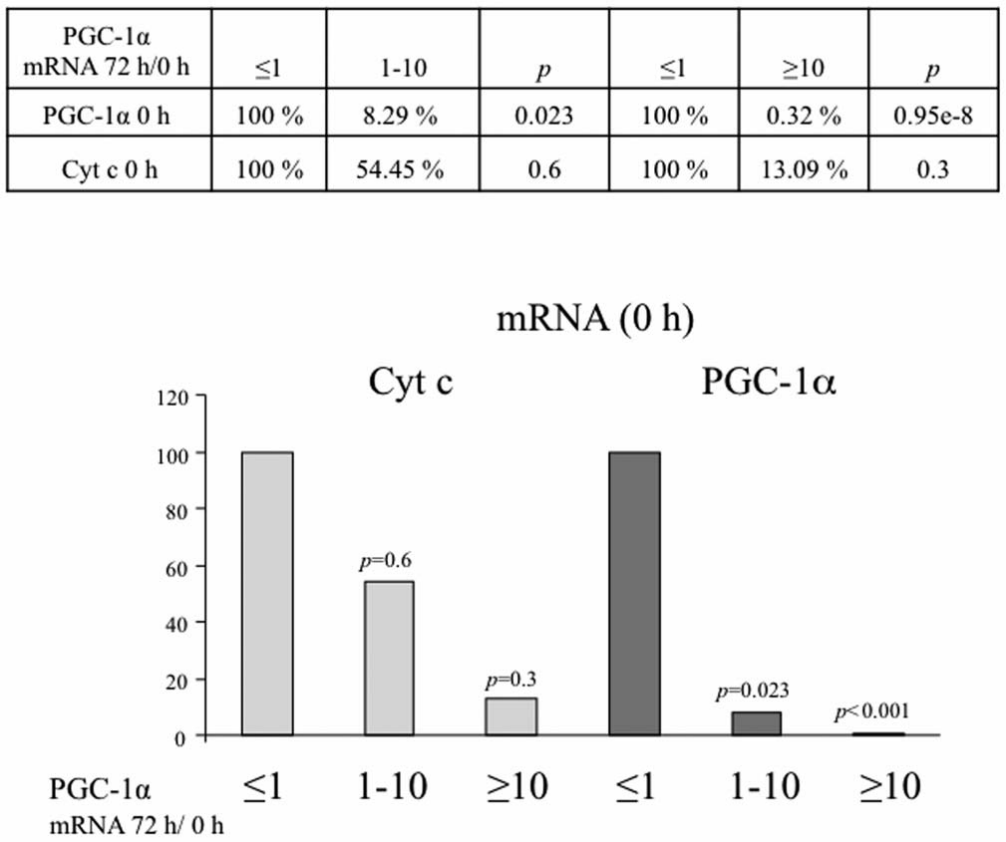
PGC-1α induction after STEMI negative correlates with PGC-1α basal levels. The graph shows average Cyt C and PGC-1α mRNA levels at the time of hospital admission in the three groups of PGC-1α induction.

Importantly, we noted that the induction of PGC-1α after STEMI inversely correlated with the mRNA levels of both PGC-1α and Cyt c at the time of admission (Fig. 3), suggesting that reduced basal PGC-1α activity, would result in stronger inductions following STEMI.

**Figure 4:**
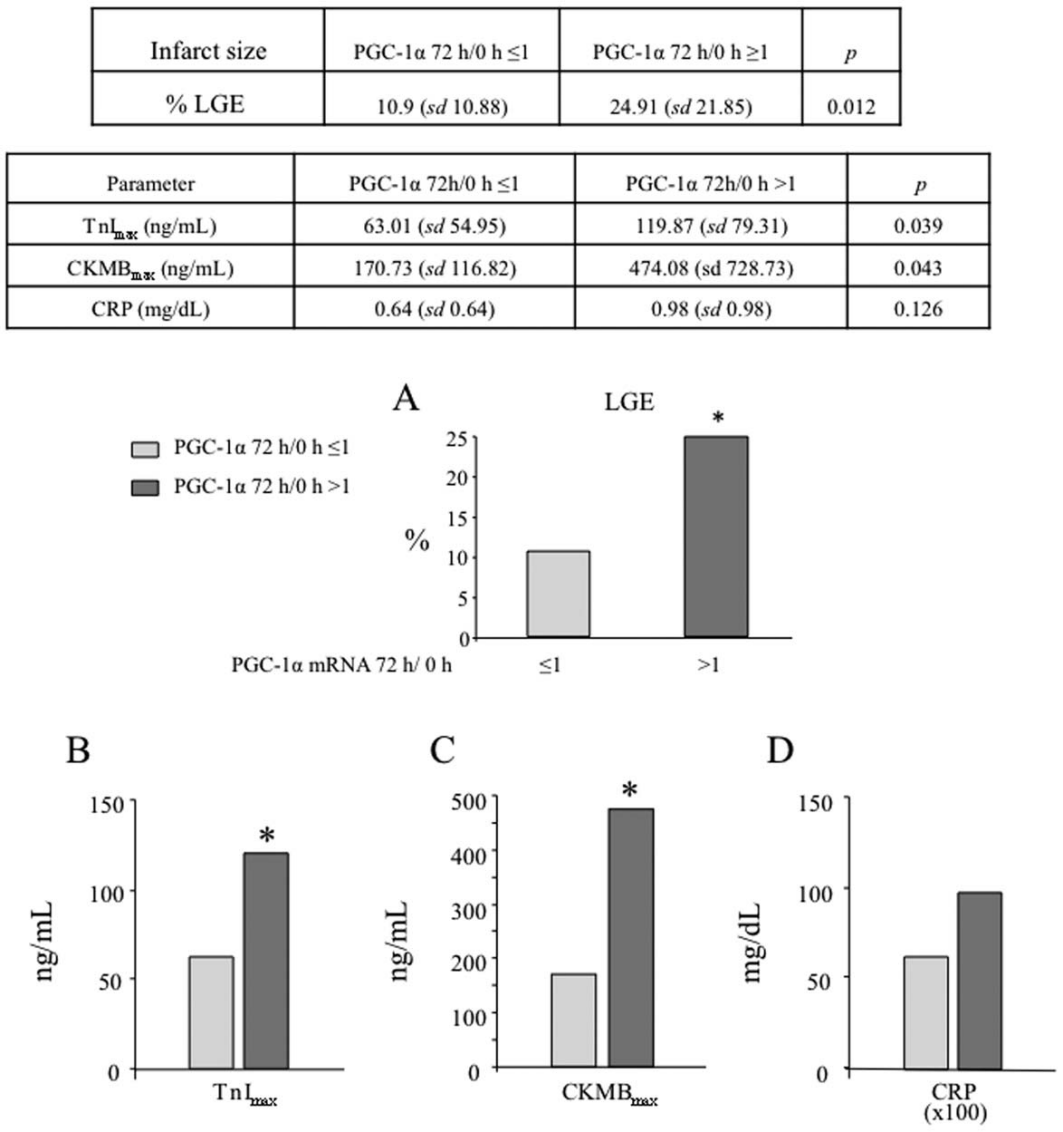
Patients that induce PGC-1α after STEMI have bigger necrotic areas. **A)** Necrotic areas as estimated by late gadolinium enhancement (LGE). The infarct sizes, of the cohort of STEMI patients under survey, was determined by LGE on cardiac RMN (CMR). The estimated mean infarct size for patients that induced PGC-1α levels (PGC-1α 72 h/0 h ≥1) is compared to that of patients that did not induce PGC-1α (PGC-1α 72 h/0 h ≤1) after STEMI. **B–D)** PGC-1α induction positively correlates with cardiac necrosis as estimated by TnI_max_ (B), CKMB_max_ (C), and CRP plasma levels (D). TnI_max_, CKMB_max_, and CRP levels of the cohort of STEMI patients under survey The estimated mean for patients that induced PGC-1α levels (PGC-1α 72 h/0 h ≥1) is compared to that of patients that did not induce PGC-1α (PGC-1α 72 h/0 h ≤1) after STEMI. Data are means +/−SD. (*) *p*<0.05.

To evaluate the functional significance of PGC-1α induction, expression of PGC-1α was compared with: blood levels of biomarkers (maximum values of TnI and CK-MB), echocardiographic parameters (LVEF and RWMA), and infarct size estimated by LGE in the CMR study. We found that induction of PGC-1α expression 72 h after STEMI correlates with bigger infarcted areas (% of necrotic area: 24.91 *vs* 10.9%) (Fig. 4A) and with higher enzymatic peak (TnI 119.87 *vs* 63.01 ng/ml and CKMB 474.08 *vs* 170.73 ng/mL) (Fig. 4B), suggesting that induction of PGC-1α expression is part of the cellular response to a STEMI.

**Figure 5:**
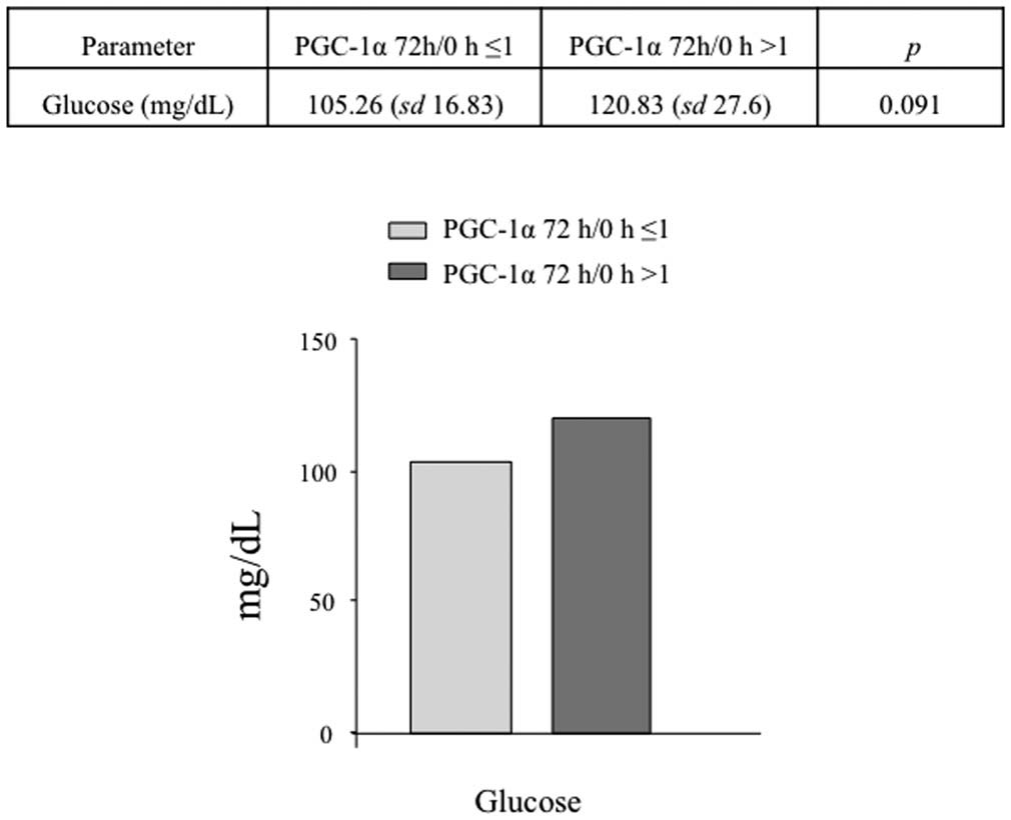
PGC-1α induction correlates with higher plasma glucose levels in the cohort of STEMI patients under survey. The estimated mean for patients that induced PGC-1α levels (PGC-1α 72 h/0 h ≥1) is compared to that of patients that did not induce PGC-1α (PGC-1α 72 h/0 h ≤1) after STEMI. Data are means +/−SD. (*) *p*<0.05.

PGC-1α induction tends to be more frequent in patients with altered glucose metabolism and correlates with higher C-reactive protein (CRP) levels, although the differences do not reach statistical significance (Fig. 4D and Fig. 5).

**Figure 6:**
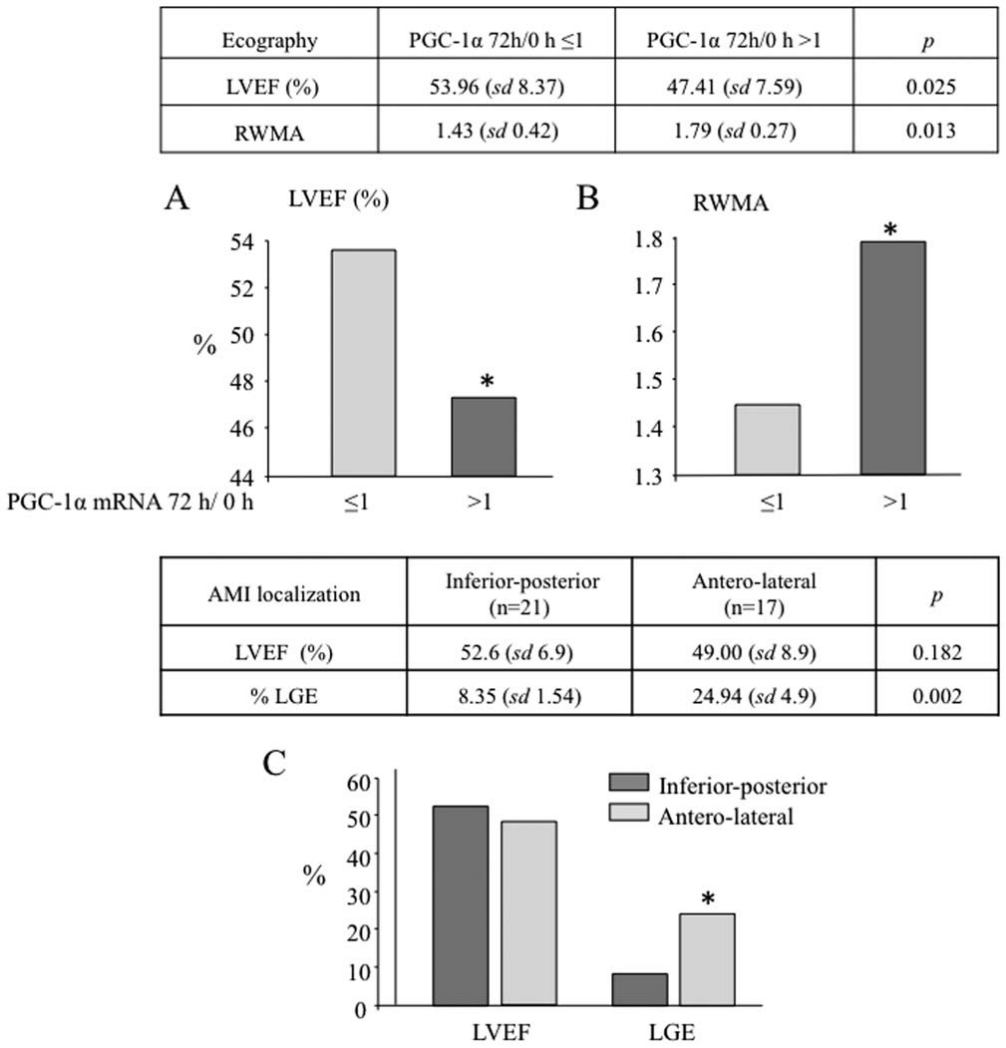
PGC-1α induction positively correlates with cardiac ventricular dysfunction. **A)** LVEF and **B)** RWMA, were determined by ecocardiography. The estimated mean for patients that induced PGC-1α levels (PGC-1α 72 h/0 h ≥1) is compared to that of patients that did not induce PGC-1α (PGC-1α 72 h/0 h ≤1) after STEMI. **C)** Antero-lateral infarcts are associated to larger necrotic areas than inferior-posterior infarcts as determine by LGE on CRM. The graph shows mean LVEF and necrotic area (LGE) of patients with antero-lateral or inferior-posterior infarcts. Data are means +/−SD. (*) *p*<0.05.

Importantly, we also found that induction of PGC-1α expression 72 h after STEMI correlates with more ventricular dysfunction, with reduced LVEF, 53.96 *vs* 47.41% (Fig. 6A), and increased RWMA (1.43 *vs* 1.79). Remarkably, left ventricular dysfunction is more frequent in patients with a strong PGC-1α induction, independently of the localization of the necrotic area and despite the bigger size of the anterior infarctions (Fig. 6C). This important observation may relate both to a stronger immune response, and to a lower systemic level of PGC-1α.

**Figure 7:**
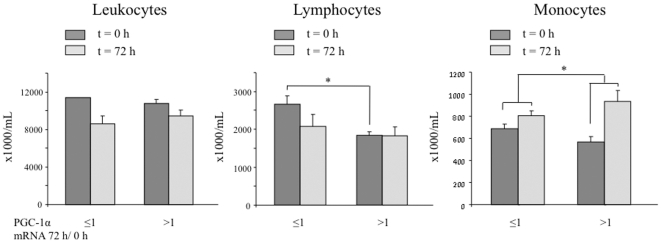
STEMI patients that induce PGC-1α do not show gross differences in the number of leukocytes. Total number of leukocytes, lymphocytes, and monocytes in blood samples of patients at the time of hospitalization (t = 0) and 72 h later. The estimated mean for patients that induced PGC-1α levels (PGC-1α 72 h/0 h ≥1) is compared to that of patients that did not induce PGC-1α (PGC-1α 72 h/0 h ≤1) after STEMI. Data are means +/−SD. (*) *p*<0.05.

In order to evaluate if the observed induction of PGC-1α monitored gross differences in the immune response after STEMI, the total and relative blood count of total leukocytes, lymphocytes and monocytes were monitored at t = 0 h and t = 72 h. As previously noted a relative lymphocyte count tends to be reduced after 72 h while the monocyte count tends to be increased. No major differences were observed between the group of patients that induced PGC-1α and the group that did not, indicating that the observed increase in PGC-1α is likely a specific induction of its expression and not just a reflection of changes in the number of leukocytes following STEMI (Fig. 7).

## Discussion

This study suggests that the monitorization of PGC-1α mRNA levels has potential interest as a novel marker for the recovery after STEMI, since high t = 0 PGC-1α levels and absence of PGC-1α induction 3 days following STEMI, strongly correlate with reduced cardiac injury. Furthermore, our data show that PGC-1α mRNA levels can be readily detected in blood mononuclear cell samples and are a good indicator of its activity.

Recovery of the cardiomyocyte oxidative capacity is crucial during the reperfusion phase after STEMI. PGC-1α is a master regulator of cellular oxidative metabolism. Since PGC-1α has been found in lymphocytes, and induction of the immune system is also a key factor in the STEMI recovery phase, we decided to evaluate if PGC-1α induction could be detected in human patients after STEMI. We found a good correlation between its post-hypoxic expression and the size of the infarcted area.

38 patients were prospectively selected with STEMI diagnostic that underwent a re-perfusion procedure. Blood samples were obtained right after their arrival to the hospital and 72 h later, being processed for RNA isolation. PGC-1α mRNA levels were analyzed by qRT-PCR. Expression of PGC-1α in blood mononuclear cells was compared with the severity of the cardiac injury estimated by: enzymatic peak, LVEF and necrotic area determined by LGE-CMR. We found that induction of PGC-1α in blood mononuclear cells positively correlates with higher levels of myocardium damage, a more severe left ventricular dysfunction, a higher RWMA score, and larger necrotic areas. This observation showed that PGC-1α induction can be detected in human blood mononuclear cells as part of the response to an STEMI, and suggested that PGC-1α is induced in the immune system (Table 1 and 2).

**Table 1:** Demographic data, Biochemistry and CMR Characteristics of the Patients with and without PGC-1α Induction.

	PGC-1α mRNA 72 h/0 h >1 (n = 12)	PGC-1α mRNA 72 h/0 h ≤1 (n = 26)	*p* value
***Base line characteristics***			
Age (years)	61.08 (*sd* 3.03)	60.88 (sd *2.33*)	0.959
Males sex (%)	10 (83.3%)	22 (84.6%)	0.924
Diabetes (%)	5 (41.7%)	8 (30.8%)	0.541
Hypertension (%)	9 (75%)	18 (69.2%)	0.722
Hypercholesterolemia (%)	6 (50%)	14 (53.8%)	0.834
Current smoker (%)	6 (50%)	15 (57.7%)	0.674
***Coronary event***			
Mean time to reperfusion (minutes)	189 (137–242)	184 (160–208)	0.942
Median troponin I peak value (ng/mL)	119.87 (97–143)	63.01 (52–74)	0.039
Median creatine kinase MB peak value (ng/mL)	474.08 (264–684)	170.73 (148–193)	0.043
Elective ACTP (%)	3 (25%)	10 (38%)	0.417
Rescue ACTP (%)	7 (58.4%)	4 (16%)	0.006
Reperfusion by thrombolysis (%)	2 (16.6%)	12 (46%)	0.084
Anterior infarction (%)	8 (66.6%)	9 (34.6%)	0.075
***Biochemistry (plasma)***			
Creatinine (mg/dL)	1.03 (0.95–1.11)	1.01 (0.97–1.05)	0.811
Haemoglobine (mg/dL)	13.10 (12.6–13.6)	13.86 (13.4–14.3)	0.249
Cholesterol (mg/dL)	177.08 (165–189)	180.92 (170–191)	0.812
LDLc (mg/dL)	116.50 (107–126)	122.19 (115–130)	0.642
HDLc (mg/dL)	38.58 (37–40)	36.23 (35–38)	0.269
Glucose (mg/dL)	120.83 (113–128)	105.26 (102–109)	0.091
CRP (mg/dL)	0.98 (0.71.1.25)	0.64 (0.57–0.71)	0.126
***Ecocardiography and magnetic resonance***			
Ejection fraction by echocardiography (%)	47.41 (*sd* 2.2)	53.96 (*sd* 1.6)	0.025
Score regional wall motion abnormalities	1.79 (*sd* 0.1)	1.43 (s*d* 0.1)	0.013
Left ventricle ejection fraction <35% (%)	16.6 (*sd* 11.2)	3.8 (*sd* 0.4)	0.136
Late gadolinium enhancement by CMR (%)	24.91 (*sd* 6.3)	10.90 (*sd* 2.1)	0.012

Data are means +/−SD. n.s = non statistically significant. Significance: *p*<0.05.

**Table 2:** White Blood Cell Subtypes of Patients with and without PGC-1α Induction.

*White blood cell subtypes*	PGC-1α mRNA 72 h/0 h ≤1 (n = 26)	PGC-1α mRNA 72 h/0 h >1 (n = 12)	*p* value
Leukocytes 0 h (×1000/mL)	11426 (*sd* 820)	10775 (*sd* 826)	n.s.
Leukocytes 72 h (×1000/mL)	8584 (*sd* 413)	9408 (*sd* 683)	n.s.
(leuko. 72 h –leuco. 0 h)/leuko. 0 h (%)	−34.3	−19.8	n.s.
Lymphocytes 0 h (×1000/mL)	2665 (*sd* 227)	1850 (*sd* 327)	0.04
Lymphocytes 72 h (×1000/mL)	2076 (*sd* 92)	1825 (*sd* 245)	n.s.
(lym. 72 h –lym. 0 h)/lym. 0 h (%)	−29.1	−5.1	n.s.
Monocytes 0 h (×1000/mL)	684 (*sd* 44)	566 (*sd* 41)	n.s.
Monocytes 72 h (×1000/mL)	807(*sd* 48)	933 (*sd* 97)	n.s.
(mono. 72 h –mono.0 h)/mono. 0 h (%)	10.9	32.1	0.05
Lymphocytes 0 h (%)	24.5 (*sd* 1.7)	18 (*sd* 3.2)	n.s.
Lymphocytes 72 h (%)	24.9 (SD +/− 1.33)	19.7 (SD +/− 2.3)	0.04
Monocytes 0 h (%)	6.2 (SD +/− 0.3)	6.1 (SD +/−0.6)	n.s.
Monocytes 72 h (%)	9.3 (SD+/− 0.3)	9.9 (SD +/− 0.5)	n.s.

Data are means +/−SD. n.s = non statistically significant. Significance: *p*<0.05.

Interestingly, PGC-1α induction tends to be more frequent in patients with altered glucose metabolism and higher C-reactive protein (CRP) levels. CRP is generally acknowledged as an important predictor of vascular death and has been shown to allow reliable risk stratification of STEMI patients [25]. Elevated PGC-1α mRNA levels have been previously reported in the liver of diabetic patients [26]. However, metabolic data in diabetic patients is generally consistent with a loss of PGC-1α activity [27]. This apparent paradox may be related to the well-established observation that diabetic patients have a chronic basal activation of the immune system [28].

Although there were no major differences in the immune response between the groups, some significant differences where observed. The induction group had significantly more lymphocytes at the time of hospitalization, and the increase in monocyte count at t = 72 h was significantly more pronounced in the induction group Although the significance of these differences requires further evaluation, it is likely that they may just be the correlate of a more severe cardiac damage.

Neutrophils, lymphocytes and monocytes are part of this response with neutrophil's count peak 12 h post-infarction, lymphocytes and monocytes 48 h post-infarction. PGC-1α has been previously found in human lymphocytes. To directly test if PGC-1α is induced in stimulated leukocytes we measured PGC-1α levels in murine splenocytes and we found that PGC-1α levels were induced following B-cell receptor (BCR) stimulation. These results support the notion that induction of PGC-1α in lymphocytes could be part of the immune activation that follows an STEMI. A possible role of PGC-1α in lymphocyte activation is supported by different observations. For example, oxygen consumption is increased in B lymphocytes following IgM activation [29], and mitochondrial activity is associated with T cell activation [30], [31], [32].

It should be noted that no changes in PGC-1α levels can be detected in murine splenocytes at times shorter than 3 h post-stimulation. Since patients with a latency time of over 150 min were excluded from the study, we believe that the values detected in human blood mononuclear cells at the time of the patient admission are a close correlate of the normal the basal levels for the patient. Nevertheless, this remains an estimate that cannot unfortunately be fixed, since it was not possible to get blood samples of the patients before the STEMI.

Overall, we interpret these results as an indication that PGC-1α expression is induced as part of the immune responsiveness following myocardial infarction in humans, and furthermore, they possibly suggest that low basal PGC-1α levels negatively correlate with cardiac damage. Following myocardial infarction an inflammatory cascade is activated and circulating total white blood cell counts increase dramatically. Larger infarct sizes and adverse outcomes correlate with white blood cell count in STEMI.

Results obtained with animal models and *in vitro* cell assays have shown that reduced blood flow and reduced oxygen tension induce PGC-1α, and conversely PGC-1α is necessary to induce angiogenesis and recover the tissue capacity to access and use oxygen, as well as to maintain muscle contraction capacity, while its absence leads to heart failure. These results, although highly informative lack so far a human correlate, mainly due to the obvious need and impossibility to take human heart samples. Our results now open a new window of possibilities since the observation that PGC-1α induction can be observed in the mononuclear cells of blood samples makes now feasible to test if PGC-1α has in fact a prognostic value for cardiac recovery.

It is widely accepted that metabolic dysfunctions, where PGC-1α levels are reduced, are an important risk factor for cardiovascular disease. Our results suggest that PGC-1α activity is also likely to be relevant in the limitation of cardiac damage following an STEMI, and that its monitorization is feasible and likely to be a relevant prognosis factor both for cardiovascular disease and for the extent of cardiac damage following STEMI.
